# Antisymmetric linear magnetoresistance and the planar Hall effect

**DOI:** 10.1038/s41467-019-14057-6

**Published:** 2020-01-10

**Authors:** Yishu Wang, Patrick A. Lee, D. M. Silevitch, F. Gomez, S. E. Cooper, Y. Ren, J.-Q. Yan, D. Mandrus, T. F. Rosenbaum, Yejun Feng

**Affiliations:** 1 Division of Physics, Mathematics, and Astronomy, California Institute of Technology, Pasadena, CA 91125 USA; 2 The Institute for Quantum Matter and Department of Physics and Astronomy, The Johns Hopkins University, Baltimore, MD 21218 USA; 3 Department of Physics, Massachusetts Institute of Technology, Cambridge, MA 02138 USA; 4 Okinawa Institute of Science and Technology Graduate University, Onna, Okinawa 904-0495 Japan; 5 The Advanced Photon Source, Argonne National Laboratory, Argonne, IL 60439 USA; 6 Materials Science and Technology Division, Oak Ridge National Laboratory, Oak Ridge, TN 37831 USA; 7 Department of Materials Science and Engineering, University of Tennessee, Knoxville, TN 37996 USA

**Keywords:** Materials science, Condensed-matter physics

## Abstract

The phenomena of antisymmetric magnetoresistance and the planar Hall effect are deeply entwined with ferromagnetism. The intrinsic magnetization of the ordered state permits these unusual and rarely observed manifestations of Onsager’s theorem when time reversal symmetry is broken at zero applied field. Here we study two classes of ferromagnetic materials, rare-earth magnets with high intrinsic coercivity and antiferromagnetic pyrochlores with strongly-pinned ferromagnetic domain walls, which both exhibit antisymmetric magnetoresistive behavior. By mapping out the peculiar angular variation of the antisymmetric galvanomagnetic response with respect to the relative alignments of the magnetization, magnetic field, and electrical current, we experimentally distinguish two distinct underlying microscopic mechanisms: namely, spin-dependent scattering of a Zeeman-shifted Fermi surface and anomalous electron velocities. Our work demonstrates that the anomalous electron velocity physics typically associated with the anomalous Hall effect is prevalent beyond the *ρ*_*xy*_(*H*_*z*_) channel, and should be understood as a part of the general galvanomagnetic behavior.

## Abstract (web summary)

Magnetoresitance (MR) is a tool to study electronic transport and spin order in metals. Here, the authors demonstrate two different microscopic origins of antisymmetric linear MR from both Zeeman-split Fermi surface and anomalous electron velocity.

## Introduction

The response of electrons to applied magnetic and electric fields has revealed the fundamental characteristics of the Fermi surface in metals, stimulated paradigms of electron correlations from Mott to Kondo, and enabled devices from spintronics^1^ to topological circuitry^2^. Historically, experiments separated the diagonal and off-diagonal components of the resistivity tensor by projecting onto forms which are symmetric or antisymmetric under field reversal. The magnetoresistance (MR), *ρ*_*xx*_(**H**), is symmetric to the magnetic field **H**; the Hall resistance, *ρ*_*xy*_(*H*_*z*_), is antisymmetric. Here we go beyond this paradigm and study the phenomenon of antisymmetric MR, an effect that is deeply entwined with ferromagnetism and demonstrated by early examples in Ni–Fe alloys with natural coercivity of a few Oe^3,4^. The intrinsic magnetization of a ferromagnet permits unusual and rarely observed manifestations of Onsager’s theorem when time reversal symmetry is broken at zero applied field^3–6^.

We first recall that Onsager’s relation for nonmagnetic materials gives electrical conductivity *σ*_*ij*_(**H**) = *σ*_*ji*_(−**H**). In an isotropic system, the electron transport equation^7^ can be expanded to linear and quadratic orders of applied electric field **E** and **H**, respectively, as the electrical current density ${\mathbf{j}} = (\sigma _{xx}^{\left( 0 \right)} + \beta H^2){\mathbf{E}}\, + \sigma _{xy}^{(0)}{\mathbf{E}} \, \times {\hat{\mathbf{H}}} + \gamma \left( {{\mathbf{E}} \cdot {\mathbf{H}}} \right){\mathbf{H}}$, where $\sigma _{xx}^{\left( 0 \right)}$ is the zero-field conductivity, $\sigma _{xy}^{(0)}$ is the Hall conductivity, and *β* and *γ* are numerical coefficients. The last term, (**E** ∙ **H**)**H**, leads to symmetric, quadratic galvanomagnetic behavior in both the MR and the planar Hall effect^8^.

In real systems, linking the observed macroscopic response to multiple microscopic effects can be difficult^9^. Experimentalists rely upon a combination of the functional forms (negative vs. positive, linear vs. quadratic, etc.) and evolution over a variable parameter space (thermal, angular, etc.)^10–12^ to identify these links. The symmetry implications of galvanomagnetic behavior can play a key role in elucidating the microscopic mechanisms at work in a range of scenarios.

In a ferromagnet, an intrinsic magnetization **M** breaks time reversal symmetry even at field **H** = 0, and Onsager’s relation takes the form *σ*_*ij*_(**H**, **M**) = *σ*_*ji*_(−**H**, −**M**)^4,9^. In this paper we treat **H** and **M** as independent and we use the term symmetric and antisymmetric to denote the response of the current to a reversal of the applied magnetic field **H**, while keeping other quantities, including **M**, fixed. Under these conditions, we identify two kinds of antisymmetric contributions to the current density:

1 $${\mathbf{j}} = A({\mathbf{M}} \cdot {\mathbf{H}}){\mathbf{E}} + B\left( {{\mathbf{M}} \times {\mathbf{E}}} \right) \times {\mathbf{H}},$$

where *A* and *B* are constants. The first term provides an antisymmetric MR. The second term, $\left( {{\mathbf{M}} \times {\mathbf{E}}} \right) \times {\mathbf{H}}$, is equivalent to $\left( {{\mathbf{M}} \cdot {\mathbf{H}}} \right){\mathbf{E}} - \left( {{\mathbf{E}} \cdot {\mathbf{H}}} \right){\mathbf{M}}$, so that it includes both the antisymmetric MR and a new term $\left( {{\mathbf{E}} \cdot {\mathbf{H}}} \right){\mathbf{M}}$. This term contributes to both *σ*_*xx*_ and *σ*_*xy*_. When **H** is confined to the *x-y* plane, we shall refer to the *σ*_*xy*_ component as the planar Hall effect^8,13^. We note that recently, both *σ*_*xx*_ and *σ*_*xy*_ components were referred to as planar angular MR of diagonal and off-diagonal types respectively^11^. In the literature, it is often assumed that **M** is aligned by **H**, in which case this term becomes even in **H**^8,13^, but here we treat the more general case.

As we demonstrate below, removing the requirement that **M** is parallel to **H** opens the door for a wide range of antisymmetric galvanomagnetic behavior, encompassing both transverse and longitudinal MR^14^ and the magnetic planar Hall effect^13^. Using a rotator apparatus and by studying magnetic materials with a large coercive field, we have been able to vary **H**, **M**, and **E** independently and distinguish a wide variety of phenomena. In this way we fully confirm the validity of the two terms in Eq. 1 in a magnetic system.

## Results

### Two microscopic mechanisms for antisymmetric linear MR

We proceed with a concrete description to demonstrate the microscopic origins of Eq. 1 in ferromagnets, starting from the transport equation for charge carriers of velocity **v** in the presence of a constant **M**, independent of **H**:

2 $$\left( {\frac{d}{dt} + \frac{1}{\tau }} \right){\mathbf{v}} = \frac{e}{{m}}{\mathbf{E}} + {\frac{{e}}{mc}}{\mathbf{v}} \times {\mathbf{H}} + \alpha {\mathbf{M}} \times {\mathbf{E}}$$

with *τ* the typical relaxation time between scattering events, *e* the electron charge, *m* the mass of the electron, *c* the speed of light, and *α* a numerical coefficient. The first two terms on the right-hand side represent the transport process driven by the externally applied field **E** and the Lorentz force, respectively, while the last term captures the anomalous Hall effect due to the presence of ferromagnetism. Solving **v** iteratively to first order in *H* for a steady state solution (Supplementary Note 1) gives

3 $${\mathbf{j}} = ne{\mathbf{v}} = \sigma _{xx}^{(0)}{\mathbf{E}} + \sigma _{xy}^{(0)}{\mathbf{E}} \times {\hat{\mathbf{H}}} + \sigma _{xy}^{({A})}{\mathbf{E}} \times {\hat{\mathbf{M}}} + \sigma _{xx}^{\left( 0 \right)}\frac{{\tau \alpha }}{c}\left( {{\mathbf{M}} \times {\mathbf{E}}} \right) \times {\mathbf{H}},$$

where $\sigma _{xx}^{(0)} = ne^2\tau /m$, $\sigma _{xy}^{(0)} = \frac{{ne^3\tau ^2}}{{m^2c}}H$, and $\sigma _{xy}^{({\mathrm{A}})} = - en\alpha \tau M$, and ${\hat{\mathbf{H}}}$ and ${\hat{\mathbf{M}}}$ are unit vectors of **H** and **M**, respectively.

The final term, $\left( {{\mathbf{M}} \times {\mathbf{E}}} \right) \times {\mathbf{H}} = \left( {{\mathbf{M}} \cdot {\mathbf{H}}} \right){\mathbf{E}} - \left( {{\mathbf{E}} \cdot {\mathbf{H}}} \right){\mathbf{M}}$, is the second term in Eq. 1. It provides a general mechanism for obtaining both antisymmetric linear MR and the planar Hall effect, which originates from the same anomalous electron velocity that underlies the ferromagnetism-induced anomalous Hall effect (the third term in Eq. 3)^15,16^. Closely related conclusions have been reached by considering the Boltzmann equation in ref. ^9^ and transport equation in ref. ^17^.

We further note that the assumption of a single *τ* and *n* in Eq. 3 breaks down in a ferromagnet^18^, which leads to an additional mechanism for inducing antisymmetric linear MR^4,6,9^. Microscopically, electrons of spin parallel (up) and antiparallel (down) to **M** have different scattering times *τ*_up_ and *τ*_dn_, with the carrier densities *n*_up_ and *n*_dn_ tuned linearly by the applied field through a Zeeman energy shift at the Fermi surface^18^. To first order in *H*, this brings a correction to *nτ* in $\sigma _{xx}^{(0)}$ where *nτ* is replaced by $n_{{\mathrm{up}}}\tau _{{\mathrm{up}}} + n_{{\mathrm{dn}}}\tau _{{\mathrm{dn}}} = ( {( {n_{{\mathrm{up}}} - n_{{\mathrm{dn}}}})( {\tau _{{\mathrm{up}}} - \tau _{{\mathrm{dn}}}}) + ( {n_{{\mathrm{up}}} + n_{{\mathrm{dn}}}})( \tau _{{{\mathrm{up}}} + \tau _{{\mathrm{dn}}}})})/2,$ with $( {n_{{\mathrm{up}}} - n_{{\mathrm{dn}}}} )\sim H.$ After projection along **M**, the first term in Eq. 3 gives a field-dependent MR that is negatively sloped and proportional to (**M** · **H**)**E** (the first term of Eq. 1). This linear form was observed in systems of saturated moments at high field^14^, and over a very narrow field range of 1–2 Oe in the hysteresis region of Ni–Fe magnets^3,4^. Since the carrier density difference is linear in *H*, this argument would introduce a second order in *H* correction to the last term (**M** × **E**) × **H** in Eq. 3 and hence is neglected here. In sum, Eq. 3 leads to the full general form given in Eq. 1.

### Experimental verification and separation of two mechanisms

Many physical systems feature large coercivities, covering a range of microscopic origins. We focus here on two such classes of materials to investigate experimentally the two antisymmetric linear forms, (**M** × **E**) × **H** and (**M** · **H**)**E**. In the rare-earth ferromagnet SmCo_5_ (and the related rare-earth ferromagnet Nd–Fe–B; see Supplementary Fig. 3), the combination of crystalline anisotropy along the *c*-axis and a needle-like microstructure^19^ gives rise to coercivities up to ±2 T at *T* = 300 K (Supplementary Fig. 1). In addition, we leverage the presence of parasitic ferromagnetism at metallic domain walls in insulating antiferromagnets^20,21^, where the large coercivity stems from the ferromagnetic moments being pinned strongly by the antiferromagnetic bulk below the Néel temperature, *T*_N_. These conditions are met by pyrochlore-structured all-in-all-out (AIAO) antiferromagnets^5,6,22,23^ such as Eu_2_Ir_2_O_7_ and Cd_2_Os_2_O_7_ (Supplementary Fig. 2), which can preserve constant ferromagnetic domain walls over a field range of at least ±14 T.

**Fig. 1:**
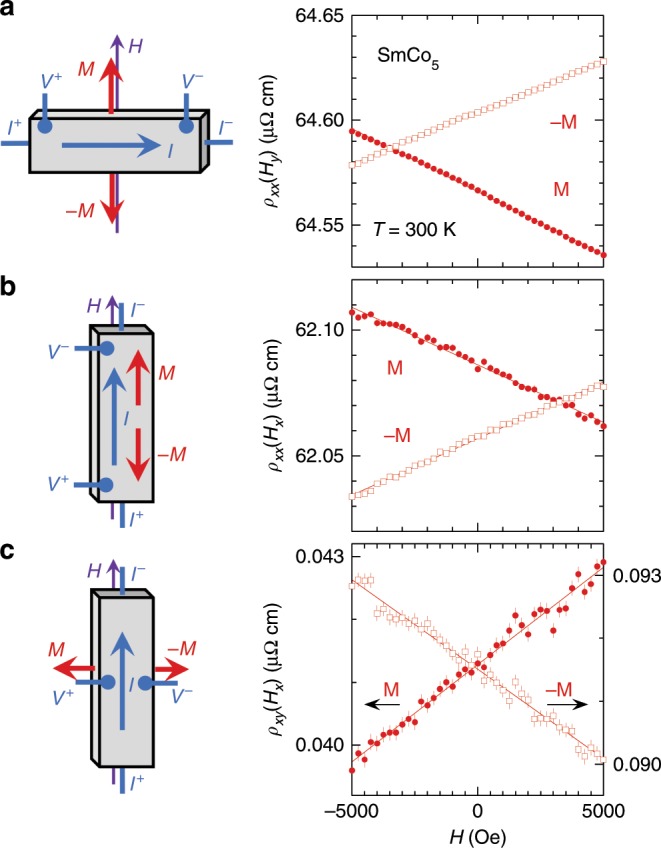
Ferromagnetism-induced antisymmetric linear magnetoresistance and the planar Hall effect. Using a fixed geometry relative to the field (schematics), *ρ*(*H*) is plotted for three types of galvanomagnetic behavior, **a** transverse MR, **b** longitudinal MR, and **c** the planar Hall effect, relative to two different magnetic states **M** and **−M** in SmCo_5_ samples. The magnetization is set by a field (±14 T) that is much larger than the measuring field (±0.5 T). In all three cases, antisymmetric linear MR of opposite slopes was observed. The two-way switching behavior rules out trivial explanations arising from imperfections in the contact geometry, and pinpoints the origin in a magnetization that is independent of the measurement field. Vertical error bars represent 1*σ* s.d. uncertainty.

We first examine the galvanomagnetic behavior of several SmCo_5_ samples in three configurations, transverse MR (Fig. 1a), longitudinal MR (Fig. 1b) and planar Hall (Fig. 1c), with both positive and negative magnetization. At *T* = 300 K, all of the *ρ*(*H*) curves manifest an antisymmetric linear behavior over a field range of ±5000 Oe with no noticeable quadratic component. The field range is chosen so that **M** varies over 15% of its fully saturated value; this range could be extended to ±1 T at *T* = 100 K with less than a 5% change in **M** (Supplementary Fig. 1). The oppositely sloped resistivity with regard to either **M** or −**M** indicates that the initial magnetization is the determining factor for the antisymmetry, rather than the trivial experimental artifact of a projected ordinary Hall component.

**Fig. 2:**
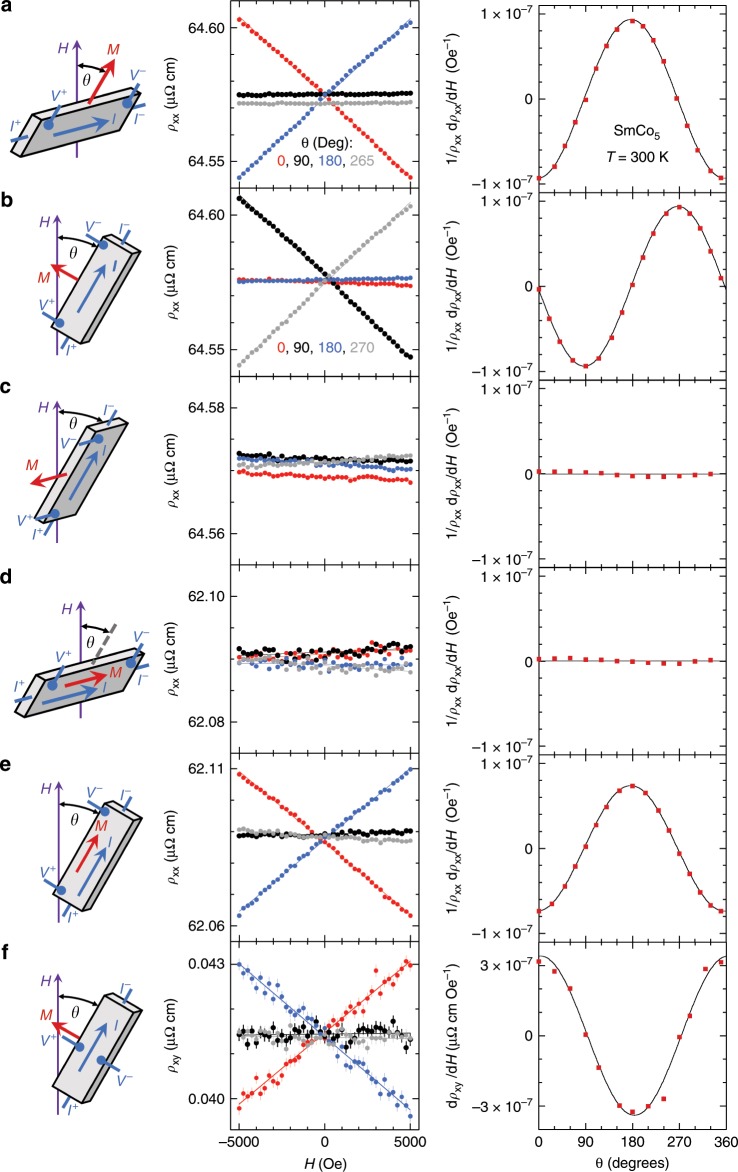
Two mechanisms of antisymmetric magnetoresistance and the planar Hall effect. With two identified mechanisms to generate the antisymmetric linear MR behavior, six rotational scenarios (**a**–**f**) were designed to create various geometries between **M**, **E**, and **H** in order to test the functional forms of both (**M** ⋅ **H**)**E** and (**M** × **E**) × **H**. In each figure, we plot schematics of the rotation scenario, raw data *ρ*(*Η*) at major angular positions, and either the normalized linear slope 1/*ρ*_*xx*_(*H* *=* 0) d*ρ*_*xx*_/d*H* or the linear slope d*ρ*_*xy*_/d*H* vs. the rotation angle *θ*. Vertical error bars represent 1*σ* s.d. uncertainty. Similar antisymmetric MR and rotation *θ*-dependence was also observed in another highly coercive magnet Nd–Fe–B (Supplementary Fig. 3).

To fully verify the functional forms (**M** × **E**) × **H** and (**M** · **H**)**E**, and to eliminate the possibility of cross-contamination between geometries, we made measurements over extended rotational degrees of freedom between all vectors **M**, **E** (as current **I**), and **H**, physically realized by a rotator stage inside our magnet/cryostat. We plot in Fig. 2 six sets of measurements, illustrating the resistive behavior of SmCo_5_ in the various **M**–**E**–**H** configurations. All of the raw *ρ*(*H*) curves are linear without discernible quadratic components, with the slopes d*ρ*(*H*)/d*H* in each configuration either null or varying sinusoidally with the rotation angle *θ*. The first four sets of angular dependence, sinusoidal in Fig. 2a, b and null in Fig. 2c, d, are consistent with both (**M** · **H**)**E** and (**M** × **E**) × **H**. However, these two terms are separable by considering the geometries shown in Fig. 2e, f. The sinusoidal form in Fig. 2e supports the presence of (**M** · **H**)**E**, as the term (**M** × **E**) × **H** vanishes because **M** is parallel to **E**. Conversely, the non-vanishing sinusoidal result in Fig. 2f provides direct proof of the (**M** × **E**) × **H** term through the planar Hall geometry, where (**M** · **H**)**E** is zero due to the orthogonal voltage and current lead configuration.

We note that contamination from other configurations is unlikely to explain the evidence for the planar Hall effect that emerges in Fig. 2f. A misalignment of the leads in the MR of Fig. 2b could provide in principle a nonzero projection of the planar Hall channel, but such a misalignment would phase shift the observed cos(*θ*) dependence toward a sin(*θ*) form. We also can rule out mixing of effects from Fig. 2e, as the signal in Fig. 2f would surpass that of Fig. 2e by two orders of magnitude when it is normalized by the residual *ρ*_*xy*_(*H* = 0). Thus, by systematically varying the relative orientations of the magnetization, the applied magnetic field, and the current, we are able to experimentally establish the presence of both physical terms in Eq. 1. Further, the (**M** × **E**) × **H** term indicates that anomalous velocity physics is not limited to the Hall channel, but instead is present in general galvanomagnetic phenomena, occurring because **M** and **H** have sequential effects on itinerant electrons. We note that in Figs. 1c and 2f anomalous electron velocity effects are fully responsible for the observed field dependence, whereas in a standard Hall geometry, the anomalous electron behavior appears in parallel with the intrinsic Hall effect. Working with a fixed **M** in the planar Hall geometry, it becomes feasible to isolate the physics of anomalous electron velocity^15,16^.

### Separation of antisymmetric linear MR from the Hall effect

The field range over which these effects can be observed is limited by the coercive field of the material. While bulk SmCo_5_ has a coercive field ~2 T at 300 K, most ferromagnetic materials have substantially lower coercive fields, ranging down to a few Oe^3,4^. An alternative approach is to consider the well-established phenomenon of parasitic ferromagnetism that exists at the interfaces of bulk antiferromagnetic domains over a very large field range^20–22^. In the AIAO antiferromagnet Cd_2_Os_2_O_7_, ferromagnetic domain walls persist over a field range of at least ±14 T. The local magnetization on each tetrahedron, either a three-in/one-out type or a two-in/two-out type, points along a local (1, 1, 1) or (1, 0, 0) axis, respectively, while magnetic domain walls exist in many orientations with minimal anisotropy^5,6,23^. Hence the ferromagnetic moment can have both parallel and perpendicular projections to the domain wall.

**Fig. 3:**
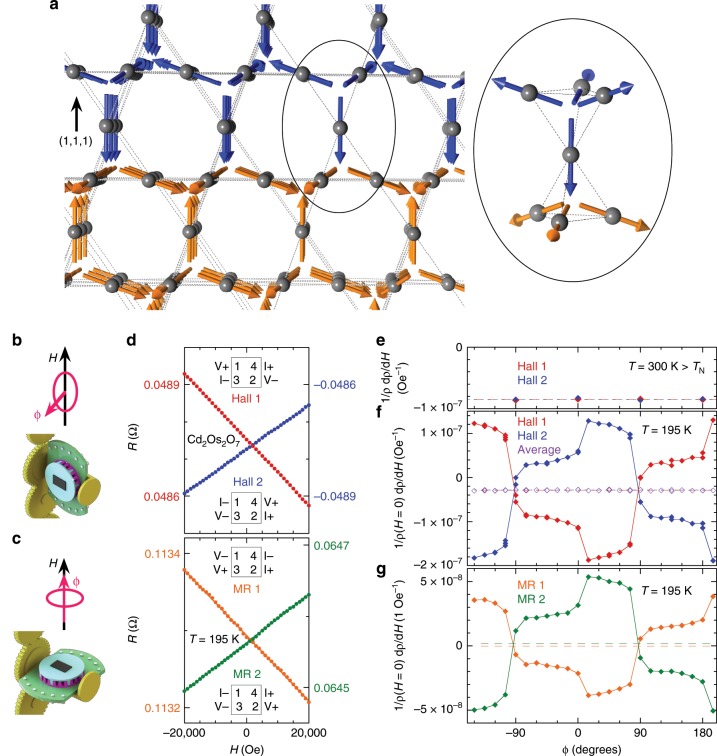
Antisymmetric linear magnetoresistance from ferromagnetic domain walls. **a** A schematic view of the domain boundary along the (1, 1, 1) plane of Cd_2_Os_2_O_7_. While the top (blue) and bottom (orange) spins make up two bulk all-in-all-out (AIAO) antiferromagnetic domains, the domain wall can be seen in this simple representation as the interfacial layer made up of individual tetrahedra with a one(blue)-in/three(orange)-out spin configuration with ferromagnetic projections perpendicular to the (1, 1, 1) plane. The inset provides a detailed three-dimensional perspective at the domain boundary. In real materials, a magnetic domain wall would form over a finite distance with spins gradually rotating in space, as exemplified by the three-in to three-out domain wall in a two-dimensional Kagome lattice^30^. **b**, **c** Schematics of a two-rotator setup, including both a motorized continuous horizontal rotator, and a 3D-printed 15-degree-increment indexing stage. A plate-shaped sample was cooled through *T*_N_ with a field-in-plane configuration (**b**), before it is flipped 90° to have the measurement field perpendicular to the surface at low temperature (**c**). The index stage sets the direction of the in-plane field cooling and magnetization. **d** Representative antisymmetric linear galvanomagnetic curves from two reciprocal Hall configurations and two independent vdP MR channels. Insets mark the lead configuration of each measurement. **e**–**g** Normalized linear slopes 1/*ρ*(*H* *=* 0) d*ρ*/d*H* as a function of in-plane **M** rotation angle *ϕ* for two Hall configurations, both above (*T* = 300 K) and below (*T* = 195 K) *T*_N_ = 227 K, and two vdP MR channels at 195 K. The *ϕ*-averaged slopes for each of four channels are marked by dashed lines of the same color. The average values of two slopes of reciprocal Hall channels at *T* = 195 K are also plotted (purple diamonds) against *ϕ*, showing no angular dependence. All error bars are smaller than symbols.

As shown in Fig. 3, ferromagnetic domain walls in antiferromagnetic Cd_2_Os_2_O_7_ (Fig. 3a) also exhibit linear antisymmetric MR (Fig. 3d), making such materials potentially more interesting for device applications. The measurements, however, are more technically complicated, as the domain walls are substantially more conductive than the bulk and thus distort the current paths. The spatial inhomogeneity provides a realization of the Parish–Littlewood mobility fluctuation model^24^, difficult to solve over a three-dimensional random domain structure^25^. With the magnetic field perpendicular to the sample surface, an imperfect four-lead geometry would also lead to a mixture of diagonal and off-diagonal terms, exacerbating the potential modeling. Here, we use a van der Pauw (vdP) measurement geometry to highlight the mixture of the antisymmetric linear galvanomagnetic behaviors of either MR or Hall origin. Furthermore, we demonstrate the separation of the ordinary Hall and magnetic MR components is possible at the macroscopic level by using the constant domain wall magnetization **M** as an independent variable.

Using a double rotation stage (Fig. 3b, c), we induced a constant ferromagnetic moment by cooling with the applied field parallel to the four-lead sample surface. After cooling below *T*_N_ = 227 K, the magnetizing field was removed and the sample rotated by 90° so **H** becomes normal to the four-lead surface (Fig. 3c). The magnetization was prepared in a series of in-plane field cooldowns from *T* = 300 K with differing orientations of the sample relative to the magnetizing field, marked by the angular variable *ϕ* (Supplementary Fig. 2). The precise origin of *ϕ* is not important, as the pairwise voltage and current leads are seldom orthogonal in the vdP configuration, and the electrical current in the vdP geometry is never parallel. Furthermore, with domain walls running through the thickness of a bulk sample, **M** contains components perpendicular and parallel to **H**, which are neither fully aligned with the field-cooling direction nor necessarily within the sample plane. It is nonetheless important to note that despite the presence of domain wall conductance, the vdP relationship at zero field (see “Methods”) is satisfied in our sample at every *ϕ* angle, establishing macroscopic homogeneity. Here we find that the measured resistance in the two independent MR channels and the two reciprocal Hall channels are all antisymmetric and linear with **H** at *T* *=* 195 K < *T*_N_ (Fig. 3d), and their slopes oscillate with a 2π period in *ϕ*. The average linear slope of the two Hall channels is *ϕ*-independent, which also equals the *ϕ*-averaged slopes of the individual channels (Fig. 3f). By comparison, the linear slopes of the Hall channels are *ϕ*-independent and identical above *T*_N_ as shown in Fig. 3e.

The two components of the linear slopes below *T*_N_ (Fig. 3f, g), i.e. the *ϕ*-dependent oscillation and the *ϕ*-independent average, can be understood through the current-voltage reciprocal relationship for the MR and Hall effect, respectively^4^. We first recall that at zero field and between two Hall configurations, *R*_43,12_(*H* = 0) = *R*_12,43_(*H* = 0) = −*R*_21,43_(*H* = 0) (Fig. 3d, lead geometries defined in inset), reflecting opposite geometric projections of the MR from unbalanced vdP channels to the two Hall channels (see “Methods” and ref.^26^). In field, both the normal Hall contribution from the antiferromagnetic bulk and the antisymmetric linear MR from domain walls with fixed **M** can contribute to the antisymmetric galvanomagnetic behavior shown in Fig. 3d. We note the antisymmetric linear MR is forbidden in the bulk AIAO antiferromagnet due to symmetry considerations^9^. As both field-antisymmetric terms are much smaller than the zero-field resistance, the measured resistance from one of the Hall channels (Fig. 3d inset) can be expressed as

4 $$R_{43,12}\left( {\mathbf{H}} \right) = R_{43,12}\left( {H = 0} \right) + R_{43,12}^{{\mathrm{wall}}}({\mathbf{H}},{\mathbf{M}}) + R_{43,12}^{{\mathrm{bulk}}}({\mathbf{H}}).$$

Here $R_{43,12}^{{\mathrm{wall}}}({\mathbf{H}},{\mathbf{M}})$ and $R_{43,12}^{{\mathrm{bulk}}}({\mathbf{H}})$ represent domain wall and bulk contributions to the antisymmetric MR and Hall effect, respectively, where both vanish at zero field. *R*_43,12_(*H* = 0) includes both bulk and domain wall contributions, and we neglect a small anomalous Hall contribution by **M** (see “Methods”), which nevertheless remains constant over the measured field range. Similar expressions could be written for the resistance of all other Hall and MR channels.

At each *ϕ* position, the measured linear slopes of two Hall configurations have exactly opposite oscillating components (Fig. 3f), which mirror the oppositely-valued *R*_43,12_(*H* = 0) and *R*_21,43_(*H* = 0) of the projected MR in the Hall channels. Indeed, taking the *ϕ*-dependent part of the linear slope as magnetoresistive in origin could explain the contrast between the two Hall channels. For domain walls with a fixed ferromagnetic moment **M** that is independent of the field **H**, the Onsager reciprocal relationship for MR would take the form of $R_{43,12}^{{\mathrm{wall}}}\left( {{\mathbf{H}},{\mathbf{M}}} \right) = R_{12,43}^{{\mathrm{wall}}}\left( { - {\mathbf{H}}, - {\mathbf{M}}} \right)$ under the vdP geometry^4,9,26^. Here, the MR contributions to linear galvanomagnetic behavior of the two Hall channels at one *ϕ* position are not reciprocal. Instead, this reciprocal relationship connects MR contributions to two Hall channels measured at *ϕ* and *ϕ* + π, respectively, i.e., where **M** is reversed between two field-in-plane cooldowns. With an antisymmetric functional form of **H**, we have $R_{43,12}^{{\mathrm{wall}}}\left( {{\mathbf{H}},{\mathbf{M}}} \right) = R_{12,43}^{{\mathrm{wall}}}\left( { - {\mathbf{H}}, - {\mathbf{M}}} \right) = - R_{21,43}^{{\mathrm{wall}}}\left( { - {\mathbf{H}}, - {\mathbf{M}}} \right) = R_{21,43}^{{\mathrm{wall}}}\left( {{\mathbf{H}}, - {\mathbf{M}}} \right)$. This explains the relative shift of phase π in the *ϕ*-dependence of the experimentally-measured linear slopes of two reciprocal Hall channels (Fig. 3f). Combined with their opposite values at each *ϕ*, these oscillating slopes follow a period of 2π, consistent with the period of *ϕ*-tuning in **M** (Fig. 3b).

In conventional Hall measurements, the voltage-current reciprocal relationship should hold between two Hall channels as $R_{43,12}^{{\mathrm{Hall}}}\left( {\mathbf{H}} \right) = R_{21,43}^{{\mathrm{Hall}}}({\mathbf{H}})$. This includes parts of the sample which either are nonmagnetic or have soft moments that follow **H**. So the fixed ferromagnetic moment **M** is an irrelevant variable, and the slopes of Hall origin are independent of *ϕ*. This includes contributions from both normal electronic Hall and anomalous Hall from antiferromagnetic and/or paramagnetic spins^27,28^, where canted spin moments vary linearly with applied field. Across the Hall and two vdP MR configurations, ratios of the *ϕ*-averaged value and oscillation amplitude of the linear slope are not constant (Fig. 3f, g), further suggesting differing origins for these two behaviors, as either a *ϕ*-independent Hall effect or a *ϕ*-oscillating MR. Using the *ϕ*-dependence of linear slopes from a series of in-plane **M**, we are able to separate macroscopically the Hall (off-diagonal) and magnetoresistive (diagonal) components of the resistivity tensor, despite both showing antisymmetric linear behavior. In Eq. 4, the summation of bulk and domain wall contributions to the antisymmetric linear galvanomagnetic behavior is verified *a posteriori*, despite the network nature of conducting domain walls inside the semi-metallic bulk.

As with the rare-earth ferromagnets, the origin of antisymmetric linear MR from the ferromagnetic domain walls of Cd_2_Os_2_O_7_ could be attributed to both (**M** · **H**) · **E** and (**M** × **E**) × **H**. Further attribution to either (**M** · **H**) · **E** or (**M** × **E**) × **H** origin is difficult, as both **M** and **E** contain components perpendicular and parallel to **H** along random domain walls. Additional clarity may be obtained by measuring thin film samples, as reducing domain wall conductance to two-dimensions should reduce the difficulty of modeling^5,24,25^.

## Discussion

As **M** follows **H** in many polycrystalline materials of negligible coercivity, the amplitude of symmetric and parabolic MR follows an angular dependence with a periodicity of π in the sin(2*θ*) functional form^13^. This functional form does not hold for single crystals due to crystalline anisotropy. In both SmCo_5_ at *T* = 300 K and Cd_2_Os_2_O_7_ at *T* *=* 195 K, traditional parabolic-shaped symmetric MR is nevertheless negligibly small (Figs. 1–3). Instead, we observed oscillations in the slopes of the antisymmetric and linear MR with a 2π period for both SmCo_5_ and Cd_2_Os_2_O_7_ (Figs. 2 and 3). The nearly perfect sinusoidal forms in SmCo_5_ (Fig. 2) are reflective of the interplay between a fixed **M** and varying **H**, while the non-sinusoidal form of Cd_2_Os_2_O_7_ in Fig. 3 reflects the crystalline anisotropy when **M** is rotated within the (1, 1, 0) plane.

The last two terms of Eq. 3, $\sigma _{xy}^{({\mathrm{A}})}{\mathbf{E}} \times {\hat{\mathbf{M}}}$ and $\sigma _{xx}^{\left( 0 \right)}\frac{{\tau \alpha }}{c}\left( {{\mathbf{M}} \times {\mathbf{E}}} \right) \times {\mathbf{H}},$ representing respectively the anomalous Hall effect and the antisymmetric linear MR, share in common the core quantity $en\alpha \tau M$ with the origin in the anomalous electron velocity. Since the strength of the $\left( {{\mathbf{M}} \times {\mathbf{E}}} \right) \times {\mathbf{H}}$ term can be determined in the planar Hall geometry (Figs. 1c, 2f), it is possible to estimate the anomalous Hall conductance from the measured antisymmetric linear MR (Supplementary Note 2). With the zero-field longitudinal conductance, $\sigma _{xx}^{\left( 0 \right)}$ ~ 1.7 × 10^4^ Ω^−1^ cm^−1^ (Figs. 1a and 2a–d), $\sigma _{xy}^{\left( {\mathrm{A}} \right)}$ is estimated to be ~7 × 10^2^ Ω^−1^ cm^−1^ in SmCo_5_ at *T* = 300 K. In the universal plot of $\sigma _{xy}^{\left( {\mathrm{A}} \right)}$ vs. $\sigma _{xx}^{\left( 0 \right)}$ with three different scaling regions for various ferromagnets (Fig. 12 of ref. ^29^), our estimated $\sigma _{xy}^{\left( {\mathrm{A}} \right)}$, together with the measured $\sigma _{xx}^{\left( 0 \right)},$ place SmCo_5_ in the intrinsic (moderately dirty) regime close to the level induced by a small impurity potential, which is expected from a highly metallic system but of commercial sintered grade. This quantitative consistency strongly supports our claim that the observed antisymmetric linear planar Hall effect is what is to be expected from the anomalous electron velocity in Eq. 3. Moreover, our Eq. 3 and the planar Hall geometry in Fig. 2f that isolates the physics of anomalous electron velocity could lead to new measurement schemes in the extrinsic (super clean metal) regime in future, where current experimental understanding is very limited^29^.

It is instructive to make a comparison of the strength of these two antisymmetric linear MR mechanisms. Both mechanisms contribute to the measured linear slopes in Fig. 2a, b, while only the mechanism of a Zeeman-split Fermi surface contributes to the measured linear slopes in Fig. 2e. We find that the amplitudes of the sinusoidal *θ*-dependences only have a small difference between ~9.3 × 10^−8^ Oe^−1^ in Fig. 2a, b, and ~7.4 × 10^−8^ Oe^−1^ in Fig. 2e. In the planar Hall geometry (Fig. 2f), the amplitude of the *θ*-dependence (~3.4 × 10^−7^ μΩ cm Oe^−1^) can be normalized by the transverse resistivity *ρ*_*xx*_ ~ 63 μΩ cm, as the induced antisymmetric linear galvanomagnetic response arises from a magnetoresistive mechanism. This yields a normalized linear slope ~0.54 × 10^−8^ Oe^−1^ due to the mechanism of anomalous electron velocity. Taken together, the ratio of strengths in SmCo_5_ of the two mechanisms, expressed as A/B in Eq. 1, is ~10.

A host of magnets with large coercivities now become candidates for leveraging antisymmetric linear MR and the planar Hall effect to elucidate microscopic physics in anomalous electron velocity related phenomena, free of concurrent ordinary Hall behavior. This should be a particularly powerful tool in understanding transport characteristics that convolute contributions from the bulk and the domain wall interfaces, such as those found at the coincident magnetic and metal-insulator phase transitions in Cd_2_Os_2_O_7_ and related iridate pyrochlores. The identification of antisymmetric MR arising solely from the metallic, ferromagnetic domain walls provides the experimental means to characterize properly the intrinsic character of the insulating, bulk antiferromagnet and its evolution with temperature and field. It in turn informs design parameters for magnetic heterostructures with pronounced spin-orbit effects.

## Methods

### Sample origins and preparation

Commercial-grade samarium-cobalt and neodymium–iron–boron magnets were purchased from the McMaster-Carr Supply Company, USA. Electrical transport samples were sliced from the bulk and polished to bar shapes (typical sizes of 5 × 2 × 0.2 mm^3^ for samarium-cobalt and 12 × 1 × 0.5 mm^3^ for neodymium–iron–boron) with no further thermal processing nor chemical modification.

Cd_2_Os_2_O_7_ single crystal samples of typical 3 × 3 × 1 mm^3^ size were grown by the vapor transport method, and possess a more significant number of grain boundaries by comparison to small octahedral-shaped crystals of 0.3–0.5 mm size^21^. After saw-dicing and polishing, plate-shaped samples were prepared with ~400 μm lateral size and 50 μm thickness, with a (1, 1, 0) surface normal.

### X-ray characterizations

Crystalline structures of all three sample systems were examined by hard x-ray (105.7 keV) diffraction at sector 11-ID-C of the Advanced Photon Source. Those samples were mounted in the x-ray transmission geometry to maximize sensitivity to their bulk properties. The diffraction data was collected by a two-dimensional Perkin Elmer amorphous silicon x-ray image plate as shown in individual Supplementary Figures.

X-ray measurements of the samarium-cobalt materials revealed a mixture of two phases, SmCo_5_ with lattice constants of *a* *=* *b* = 5.0122 Å, *c* = 3.9774 Å, and Sm_5_Co_19_ with *a* *=* *b* = 5.0387 Å, *c* = 48.4955 Å. Molar percentages of the two phases are ~90% and ~10%, respectively. Both constituents are of hexagonal structure with the *c*-axis aligned along the magnetization direction, spanning a ±10-degree (FWHM) mosaicity (Supplementary Fig. 1).

### Magnetic hysteresis

Magnetization measurements of Cd_2_Os_2_O_7_ and SmCo_5_ were performed respectively in a 7 T Magnetic Property Measurement System (MPMS3, Quantum Design) based on a DC Superconducting Quantum Interference Device. All samples were mounted on quartz half cylinders with rotational angles determined from photographic images. The Cd_2_Os_2_O_7_ sample was field cooled under either a +4 or −4 Tesla field to *T* = 195 K before the hysteresis measurement.

### Electrical transport measurements

Electrical transport measurements on three bar-shaped SmCo_5_ samples, were performed using the usual four-probe geometry with a Lakeshore LS372 ac resistance bridge and a 3708 preamplifier for metallic samples. All SmCo_5_ samples were mounted on an 8-pin DIP connector using Stycast 2850 black epoxy to prevent sample rotation under the torque from the magnetic field. Each packaged sample was further mounted on the chip carrier of a motorized horizontal rotator stage (4084–304, Quantum Design) inside a 14 T PPMS DynaCool. All samples typically were magnetized by a 14 T field at 300 K along their natural *c*-axis before the angular dependence study was performed. MRs were measured with tens to hundreds of measurements at each magnetic field, with the field evenly stepped and cycled in a loop between the positive and negative limits in order to achieve sufficient statistics. There was no noticeable resistance hysteresis nor change of initial magnetization after all the angular positions were studied. No symmetric component is noticeable in the raw data in any geometry, given that the parabolic-shaped symmetric MR is negligibly small at 300 K despite ~15% of the total magnetic moment following the applied field (Supplementary Fig. 1c, d). All resistivity measurements presented in this work are thus the averaged raw data at each field without any antisymmetrization. The change of magnetization from **M** to **−M** causes small changes in *ρ*(*H* = 0) (Fig. 1) due to rearrangement of magnetic domains. For the sample in the planar Hall geometry (Figs. 1c and 2f), voltage leads were Pb soldered and the contact areas were carefully shaved by a sharp razor to achieve a minimized *ρ*_*xy*_(*H* = 0) and avoid projection from a transverse MR.

Electrical transport measurements on Nd–Fe–B, similarly in a long bar shape of four-probe geometry, were performed using a Linear Research LR700 resistance bridge in a 9 T PPMS DynaCool. Instead of a rotator stage, differently wedged blocks were used to vary angles between **M** and **H**. The transport sample was measured under its original magnetization condition, after the magnetic hysteresis property was studied on a separately prepared (shorter) sample. As shown in Supplementary Fig. 3, only the configuration of Fig. 2a was studied to verify Eq. 1.

Cd_2_Os_2_O_7_ samples were wired in a vdP geometry for galvanomagnetic measurements using a Lakeshore LS372 ac resistance bridge and a 3708 preamplifier. The sample circuit was mounted with two rotational degrees of freedom, one provided by the horizontal rotator option of the Quantum Design PPMS, the second provided by a home-built, 3D-printed miniature indexing stage with 24 angular positions at 15-degree steps (Fig. 3b, c). In the vdP measurement geometry, the vdP relationship at zero field (Δ_vdP_ = *R*_MR1_ − *R*_MR2_ − *R*_Hall_ = *R*_24,31_ − *R*_41,23_ − *R*_43,12_ = 0) is satisfied at the macroscopic level in our sample to a level that Δ_vdP_/*R*_MR1_ < 7 × 10^−4^ (Fig. 3d). There does exist a very small difference at zero field between two Hall channels Δ*R*_Hall_/*R*_Hall_ = (*R*_43,12_ + *R*_21,43_)/*R*_43,12_ ~ 5 × 10^−4^ due to a finite anomalous Hall effect from the fixed **M**.

## Supplementary information

Supplementary Information

## Data Availability

The data that support the findings of this study are available from the corresponding authors upon request.
