# Supplementary material for: Antisymmetric linear magnetoresistance and the planar Hall effect
Source: Nat Commun. 2020 Jan 10;11:216. doi: 10.1038/s41467-019-14057-6 (PMC6954222; doi:10.1038/s41467-019-14057-6)
Supplement: Supplementary file 1 — Supplementary Information [file 41467_2019_14057_MOESM1_ESM.pdf]

Supplementary Materials:

Antisymmetric linear magnetoresistance and the planar Hall effect

Yishu Wang[1,2], Patrick A. Lee[1,3], D. M. Silevitch[1], F. Gomez[1], S. E. Cooper[4], Y. Ren[5], J.-Q. Yan[6], D. Mandrus[6,7], T. F. Rosenbaum[1,*], Yejun Feng[1,4,*]

[1]Division of Physics, Mathematics, and Astronomy, California Institute of Technology, Pasadena, California 91125, USA

[2]The Institute for Quantum Matter and Department of Physics and Astronomy, The Johns Hopkins University, Baltimore, Maryland 21218, USA

[3]Department of Physics, Massachusetts Institute of Technology, Cambridge, Massachusetts 02138, USA

[4]Okinawa Institute of Science and Technology Graduate University, Onna, Okinawa 904-0495, Japan

[5]The Advanced Photon Source, Argonne National Laboratory, Argonne, Illinois, 60439, USA

[6]Materials Science and Technology Division, Oak Ridge National Laboratory, Oak Ridge, Tennessee 37831, USA

[7]Department of Materials Science and Engineering, University of Tennessee, Knoxville, Tennessee 37996, USA

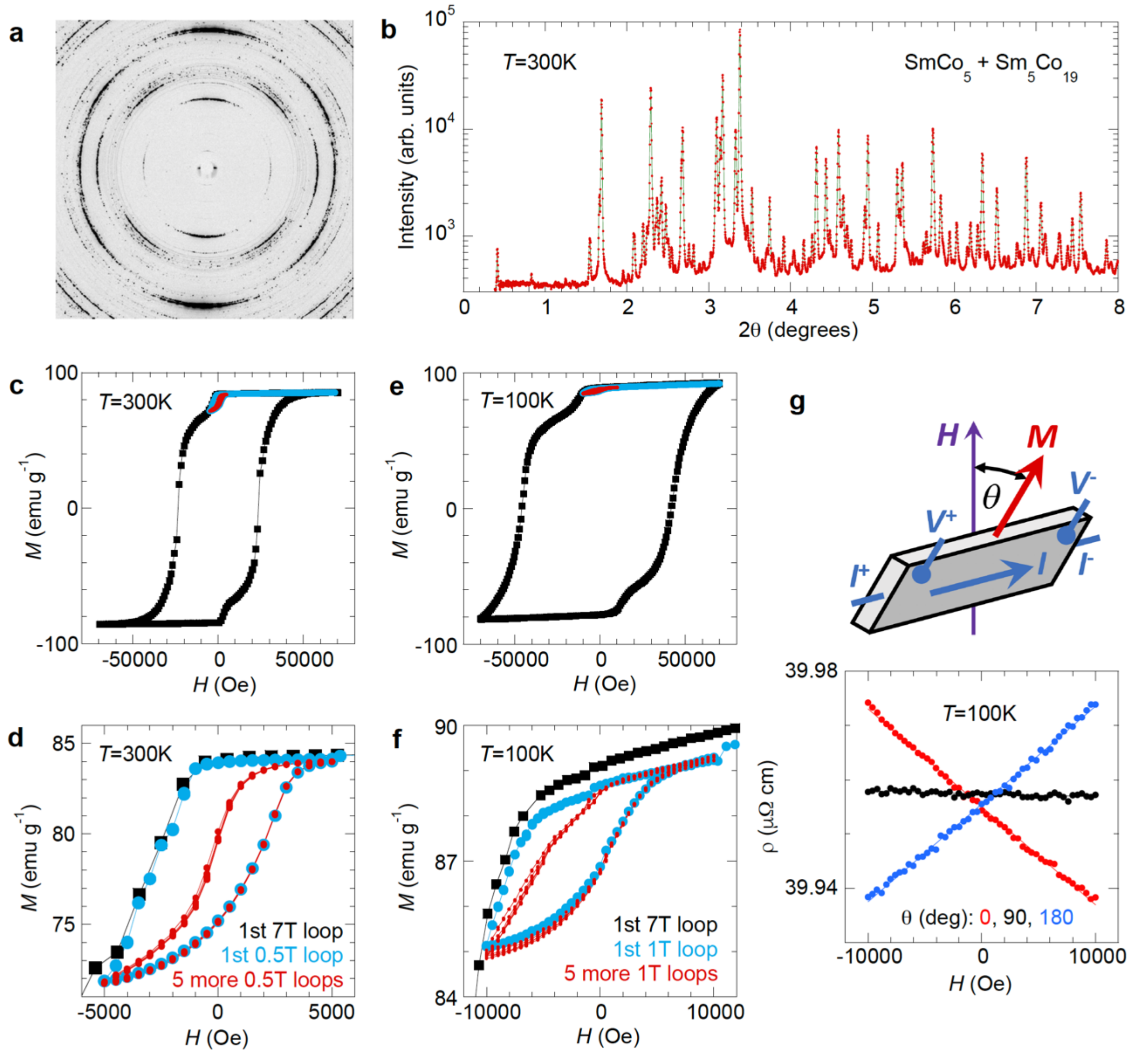

Supplementary Fig. 1. Structural and magnetic characteristics of SmCo. (**a**) Hard x-ray (105.7 keV) diffraction image of one of the transport samples. The diffraction data indicate an oriented polycrystal with the *c*-axis aligned to within ±10° FWHM mosaic range. Diffraction data also indicate that the alignment is random in the *a*-*b* plane of the tetragonal structure. (**b**) Radially integrated intensity of the diffraction pattern reveals coexistence of two phases, $SmCo_5$ and Sm_5Co_{19}. Due to the textured structure, only unit cell parameters are refined. (**c-d**) Magnetic hysteresis of the SmCo sample with magnetic field applied along the *c*-axis at T = 300 K. Black symbols mark the overall ±7 T hysteresis loop, and the blue and red symbols indicate the first and five subsequent hysteresis loops of ±0.5 T range, respectively. (**e-f**) Magnetization study similar to (**c-d**) but at T = 100 K with a 1 T loop. As the sample is field cooled under 7 T at 100 K, it is sufficiently hysteretic that a -7 T field cannot fully reverse the magnetization. (**g**) The asymmetric linear MR was observed over a ±1 T range at T = 100 K when the relative directions of **M** and **H** are varied by angle θ.

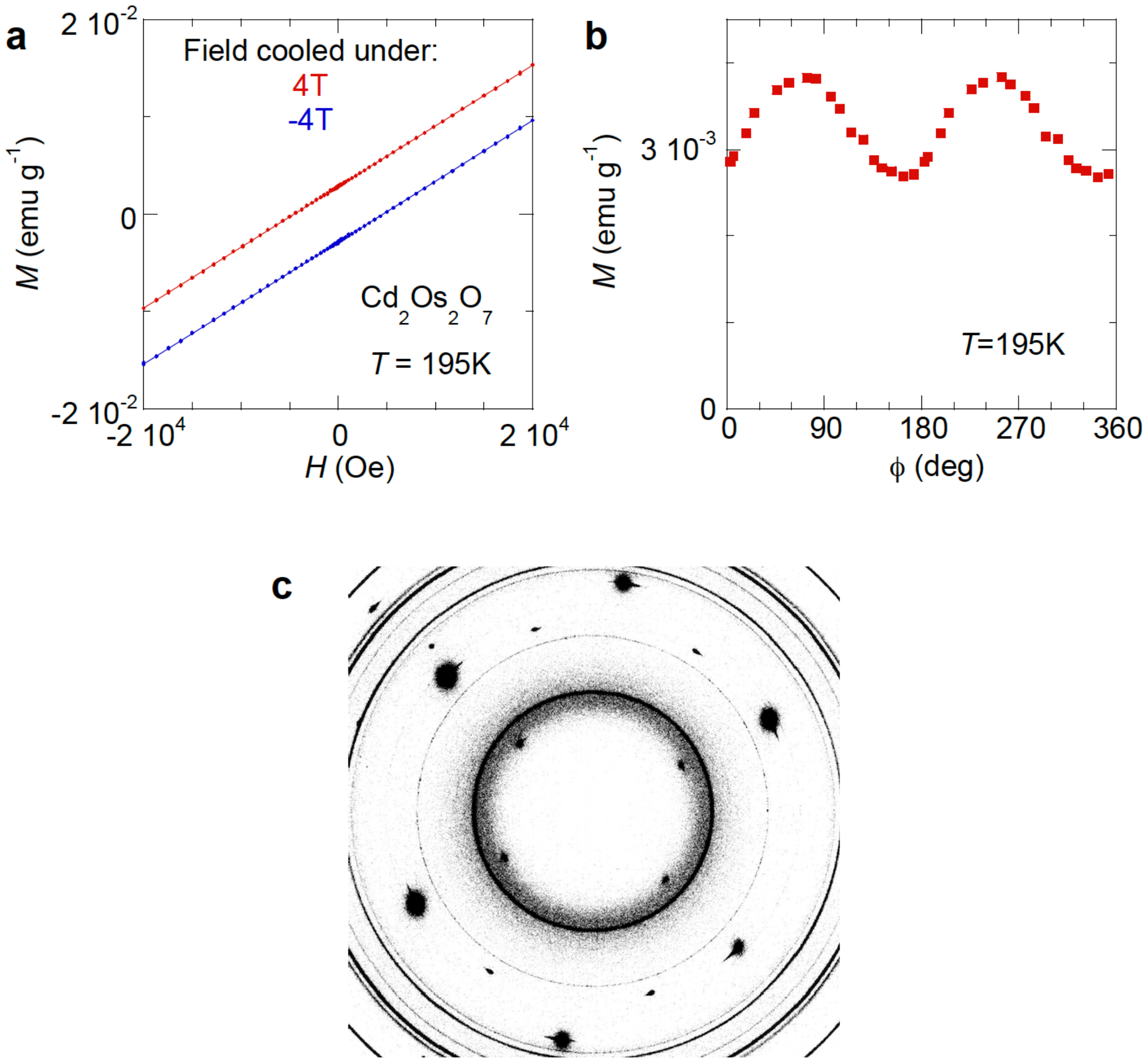

Supplementary Fig. 2. Magnetic and structural characteristics of single crystal $Cd_2Os_2O_7$. (**a**) For samples that are field cooled (4 T) through T_N, the magnetization (red) as a function of field is not hysteretic, but has a finite intercept at zero field. The frozen moment **M** changes sign when the field direction is reversed to -4 T in a separate cooldown (blue). (**b**) For a piece of $Cd_2Os_2O_7$ single crystal with a surface normal of (1, 1, 0), the zero-field frozen magnetization **M**(ϕ) was measured under various field cooldowns with a field-in-plane geometry of different ϕ. A finite **M** is always measured along the field direction, while the magnitude varies. (**c**) Hard x-ray (105.7 keV) diffraction image of the transport sample, showing the (1, 1, 0) zone with a two-fold symmetry. The spread of diffraction spots indicates a finite mosaic structure. Continuous circles are from x-ray diffraction of the rotator sample holder.

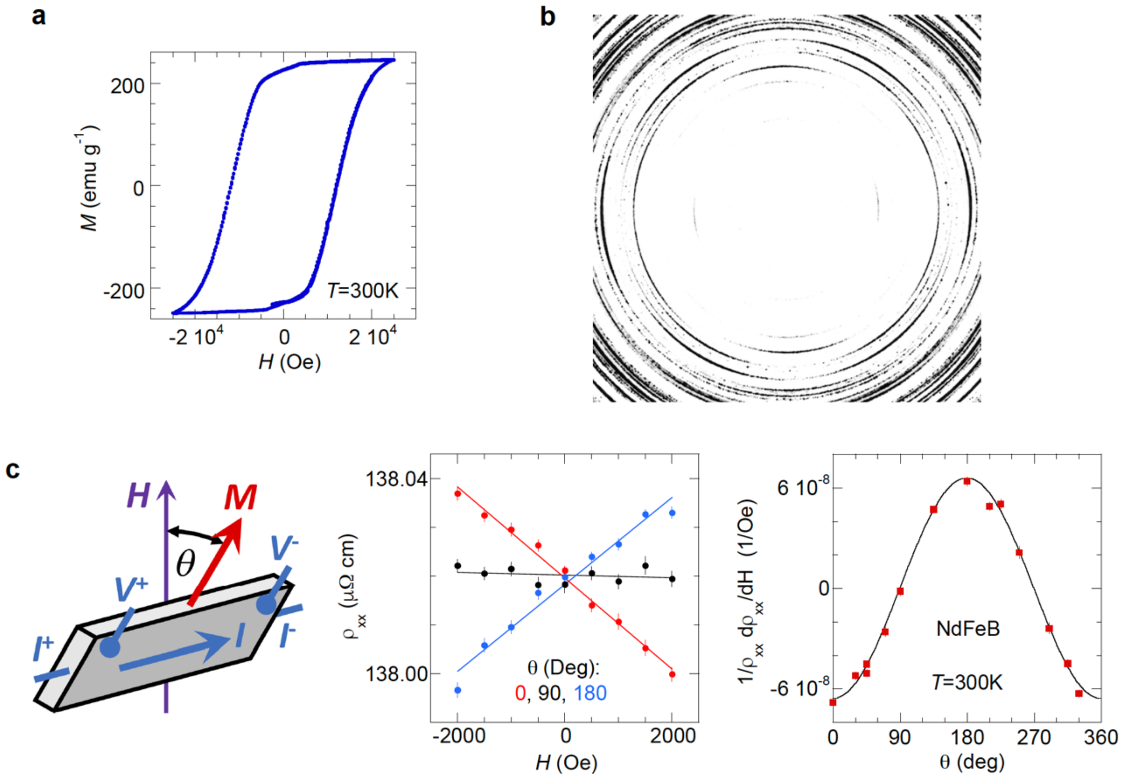

Supplementary Fig. 3. Structure, magnetic hysteresis, and antisymmetric linear MR in Nd-Fe-B magnets. (**a**) Magnetic hysteresis of the Nd-Fe-B sample along its easy axis. (**b**) Hard x-ray (105.7 keV) diffraction image of the transport sample. The diffraction data indicate an oriented polycrystal with the *c*-axis aligned to within ±15° FWHM mosaic range. (**c**) The highly coercive ferromagnet Nd-Fe-B demonstrates antisymmetric linear MR behavior similar to that of $SmCo_5$. The rotation test between **M**, **E**, and **H** is consistent with the scenario demonstrated in Fig. 2a, verifying both the (**M**·**H**)**E** and (**M**×**E**)×**H** terms. Vertical error bars represent 1σ s.d. uncertainty.

Supplementary Note 1: Derivation of Eq. 3 in the main text.

The transport equation for charge carriers in the presence of a constant magnetization **M** takes the following form of Eq. 2 in the main text:

$$(\frac{d}{dt}+\frac{1}{\tau})\mathbf{v}=\frac{e}{m}\mathbf{E}+\frac{e}{mc}\mathbf{v}\times\mathbf{H}+\alpha\mathbf{M}\times\mathbf{E}, \tag{1}$$

In a steady state, the time derivative $\frac{d\mathbf{v}}{dt}$ vanishes.

$$\frac{\mathbf{v}}{\tau}=\frac{e}{m}\mathbf{E}+\frac{e}{mc}\mathbf{v}\times\mathbf{H}+\alpha\mathbf{M}\times\mathbf{E}. \tag{2}$$

To solve **v** iteratively to the first order of H, we first keep the zeroth order of H on the right-hand side,

$$\frac{\mathbf{v}}{\tau}=\frac{e}{m}\mathbf{E}+\alpha\mathbf{M}\times\mathbf{E}+O(H). \tag{3}$$

Inserting Eq. 3 on the right side of Eq. 2 yields:

$$\frac{\mathbf{v}}{\tau}=\frac{e}{m}\mathbf{E}+\alpha\mathbf{M}\times\mathbf{E}+\frac{e}{mc}\tau\left(\frac{e}{m}\mathbf{E}+\alpha\mathbf{M}\times\mathbf{E}\right)\times\mathbf{H}. \tag{4}$$

Therefore, we get the steady state solution of **v**:

$$\mathbf{v}=\frac{e\tau}{m}\mathbf{E}+\alpha\tau\mathbf{M}\times\mathbf{E}+\frac{e^2\tau^2}{m^2c}\mathbf{E}\times\mathbf{H}+\frac{e\alpha\tau^2}{mc}\mathbf{M}\times\mathbf{E}\times\mathbf{H}. \tag{5}$$

Correspondingly, the current density **j** is:

$$\mathbf{j}=ne\mathbf{v}=\frac{ne^2\tau}{m}\mathbf{E}+\frac{ne^3\tau^2}{m^2c}\mathbf{E}\times\mathbf{H}+en\alpha\tau\mathbf{M}\times\mathbf{E}+\frac{ne^2\alpha\tau^2}{mc}\mathbf{M}\times\mathbf{E}\times\mathbf{H}. \tag{6}$$

This is Eq. 3 in the main text:

$$\mathbf{j}=\sigma_{xx}^{(0)}\mathbf{E}+\sigma_{xy}^{(0)}\mathbf{E}\times\hat{\mathbf{H}}+\sigma_{xy}^{(\mathrm{A})}\mathbf{E}\times\hat{\mathbf{M}}+\sigma_{xx}^{(0)}\frac{\tau\alpha}{c}(\mathbf{M}\times\mathbf{E})\times\mathbf{H}, \tag{7}$$

where $\sigma_{xx}^{(0)}=\frac{ne^2\tau}{m}, \sigma_{xy}^{(0)}=\frac{ne^3\tau^2}{m^2c}H$ and $\sigma_{xy}^{(\mathrm{A})}=-en\alpha\tau M$, representing conductance from normal electronic scattering, the ordinary Hall effect, and the anomalous Hall effect, respectively.

Supplementary Note 2: Estimation of anomalous Hall conductance from the planar Hall effect.

We first specify the coordinate system in Fig. 1c for the planar Hall measurement. We take $\mathbf{E}$ (as indicated by current I) applied along $\hat{\mathbf{x}}$, $\mathbf{j}$ (as selectively measured by voltage V) set along $\hat{\mathbf{y}}$ for the planar Hall effect, and $\hat{\mathbf{z}} = \hat{\mathbf{x}} \times \hat{\mathbf{y}}$. With $\mathbf{M}$ along $\hat{\mathbf{y}}$, Eq. 3 of the main text becomes:

$$\mathbf{j} = \sigma_{xx}^{(0)}\mathbf{E} + \sigma_{zx}^{(\mathrm{A})}\mathbf{E} \times \widehat{\mathbf{M}} + \sigma_{yx}^{(\mathrm{PHE})}\left(\widehat{\mathbf{M}} \times \mathbf{E}\right) \times \mathbf{H}, \quad (8)$$

where $\sigma_{xx}^{(0)} = \frac{ne^2\tau}{m}$, $\sigma_{zx}^{(\mathrm{A})} = -en\alpha\tau M$, $\sigma_{yx}^{(\mathrm{PHE})} = \sigma_{xx}^{(0)}\frac{\tau\alpha}{c}M$ are zero-field longitudinal conductance, anomalous Hall conductance, and planar Hall conductance, respectively. Noting that $\sigma_{zx}^{(\mathrm{A})}$ and $\sigma_{yx}^{(\mathrm{PHE})}$ share the core component $en\alpha\tau M$, j_y thus takes the following form:

$$j_y^{(\mathrm{PHE})} = \frac{\tau\alpha M}{c}\sigma_{xx}^{(0)}E_xH_x = \frac{1}{nec}\sigma_{xx}^{(0)}\sigma_{zx}^{(\mathrm{A})}E_xH_x. \quad (9)$$

Therefore,

$$\sigma_{yx}^{(\mathrm{PHE})} = \frac{1}{nec}\sigma_{xx}^{(0)}\sigma_{zx}^{(\mathrm{A})}H_x. \quad (10)$$

Experimentally, we measured resistivity ρ instead of conductivity σ. Assuming that the MR effect is small ($\rho_{xx} \approx \rho_{xx}^{(0)}$) and the zero-field resistivity tensor is nearly isotropic ($\rho_{xx}^{(0)} = \rho_{yy}^{(0)}$), ρ and σ are related by:

$$\sigma_{yx} = \rho_{yx}\left(\sigma_{xx}^{(0)}\right)^2, \sigma_{xx} = \frac{1}{\rho_{xx}}, \quad (11)$$

Combining Eq. 10 and Eq. 11 yield:

$$\rho_{yx}^{(\mathrm{PHE})} = \sigma_{yx}^{(\mathrm{PHE})}\left(\rho_{xx}^{(0)}\right)^2 = \frac{1}{nec}\rho_{xx}^{(0)}\sigma_{zx}^{(\mathrm{A})}H_x. \quad (12)$$

Eq. 12 naturally provides an estimate of $\sigma_{zx}^{(\mathrm{A})}$ using the slope of $\rho_{yx}^{(\mathrm{PHE})}$ vs. H:

$$\frac{d\rho_{yx}^{(\mathrm{PHE})}}{dH_x} = \frac{1}{nec}\rho_{xx}^{(0)}\sigma_{zx}^{(\mathrm{A})}. \quad (13)$$

Therefore, the anomalous Hall conductance is determined by:

$$\sigma_{zx}^{(\mathrm{A})} = nec\left(\rho_{xx}^{(0)}\right)^{-1}\left(\frac{d\rho_{yx}^{(\mathrm{PHE})}}{dH_x}\right). \quad (14)$$

Our $SmCo_5$ samples have $\rho_{xx}^{(0)} \sim 63\ 10^{-6}\ \Omega$ cm at $T = 300$ K (Figs. 1, 2), and $\frac{d\rho_{yx}^{(\mathrm{PHE})}}{dH_x} = 3\ 10^{-13}$ Ω cm Oe^{-1}. We also note the conversion between SI and Gaussian cgs units, which gives $\frac{\rho_{\mathrm{G}}}{\rho_{\mathrm{SI}}} = \frac{1\mathrm{s}}{9\times10^9\Omega\ \mathrm{m}}, \frac{H_{\mathrm{G}}}{H_{\mathrm{SI}}} = \frac{4\pi\times10^{-3}\ \mathrm{Oe}}{1\ \mathrm{A/m}}, \frac{q_{\mathrm{G}}}{q_{\mathrm{SI}}} = \frac{9\times10^9\mathrm{Fr}}{1\mathrm{C}}$, with q the electrical charge. We further assume that carrier density n in $SmCo_5$ is about $\sim 10^{22}\ \mathrm{cm}^{-3}$, as is similar for most metallic elements. With

all numerical values included in Eq. 7, we estimate the anomalous Hall conductance in $SmCo_5$ to be $\sigma_{zx}^{(A)} = 7.6 \times 10^2\ \Omega^{-1}\ cm^{-1}$.
